# Characterization of virulence factors and antibiotic resistance pattern of uropathogenic *Escherichia coli* strains in a tertiary care center

**DOI:** 10.12688/f1000research.125596.2

**Published:** 2022-11-28

**Authors:** Naveen Kumar M, Sevitha Bhat, Archana Bhat K, Vishwas Saralaya, Shalini Shenoy Mulki

**Affiliations:** 1 Department of Microbiology, Kasturba Medical College, Manipal Academy of Higher Education, Manipal, Manipal, India

**Keywords:** Virulence factors, Uropathogenic Escherichia coli, genes, antibiotic resistance, multiplex PCR

## Abstract

**Background: **Urinary tract infection(UTI) is one of the commonly prevalent bacterial infection in humans.The uropathogenic *E. coli* (UPEC) expresses a range of virulence factors that contribute to their pathogenicity *. *The emergence of multidrug resistance (MDR)-associated UTI is increasing.This study monitors the distribution of virulence factors among UPEC strains to note the antibiogram, outcome and type of associated UTI.

**Methods:**A prospective cross-sectional time-bound study of six months was done on clinically significant urinary isolates of *Escherichia coli. *Detection of haemolysin production and serum resistance was done by phenotypic methods. Genotypic characterization of the virulence genes ( *pap*C, *iut*A, *hly*A, *cnf*1) was done by multiplex PCR. Demographic data, clinical history, antibiogram and type of UTI was collected from clinical case records.

**Results:**75 *E.coli* isolates from patients with suspected UTIs were included. Females had a higher preponderance of UTI (66.7%). 93% of patients were adults and the remaining 7% were from paediatrics.  24 (32%) isolates showed haemolysis by plate haemolysis and all isolates were serum-resistant. Out of 75 isolates, 65 were positive for at least one of four targeted genes, while remaining ten isolates were negative for all four genes.Multidrug resistance was found in 40 (53.3%) isolates. 97.4% of the UTI cases had a favourable clinical outcome at discharge. Mortality due to urosepsis was 2.6%.

**Conclusion:**Association of hemolysin production with resistance to imipenem and norfloxacin in UPEC strains was significant.Presence of *hlyA *gene is positively associated with ceftazidime resistance. Nitrofurantoin, piperacillin, tazobactam, and cefaperazone sulbactam are possible candidates for empirical therapy of UTIs. Drugs like aminoglycosides, carbapenems and fosfomycin may be used as reserve drugs in the treatment of MDR-UTI.However, inappropriate usage can increase antibiotic resistance. Hence proper selection of antibiotics in hospitals taking into account the local antibiogram is needed to reduce the emergence of antibiotic resistance.

## Introduction

Background: Urinary tract infection (UTI) is one of the most prevalent bacterial infection in humans. Every year, globally about 150 million people are diagnosed with UTI. ^1^ *Escherichia coli*, *Klebsiella* spp., *Pseudomonas aeruginosa, Proteus mirabilis, Citrobacter* spp., *Staphylococcus* spp., *Enterococcus* spp., are the most common species causing UTI. *Escherichia coli* is the most frequent pathogen in the human urinary tract and accounts for 75% of the UTI. 85% of the community acquired UTIs and 40% of the hospital acquired UTI are attributed to *Escherichia coli.* ^2^

The term uropathogenic *E. coli* (UPEC) refers to the extraintestinal strains of *E. coli* that express a variety of virulence factors, contributing to their pathogenicity in comparison to commensal *E. coli.* ^3^^,^^4^ The specific virulence genes present in strains of UPEC isolates are the genes that encode adhesins (e.g. Type I fimbriae and P fimbriae), mechanisms for acquisition of nutrients (e.g. siderophores), factors that help the UPEC to escape from host defence systems (e.g. lipopolysaccharide, capsule) and toxins (e.g., cytotoxic necrotising factor 1, hemolysin). ^3^ The factors mentioned above equip the bacteria with the ability to colonize the periurethral region, ascend the urinary tract to reach the urinary bladder resulting in infections like cystitis, urethritis, pyelonephritis, and urosepsis. ^5^ UPEC strains have acquired the virulence genes (chromosomal or plasmid mediated by horizontal transfer of DNA). The molecular based platforms aid in the detection and characterization of UPEC strains. ^4^ The characterization of these virulence genes in UPEC strains will help in better understanding of pathogenesis and course of UTI. Based on previously published literature, ^3^^,^^4^ we aimed to look into the common genes *pap*C and *iut*A and apart from them, other genes *hly* A and *cnf*1 were screened for epidemiological and virulence characterisation.

Recently there is an upsurge in UPEC strains that are multidrug-resistant (MDR), i.e., resistant to at least three or more classes of antibiotic agents. ^6^ The emergence of MDR-associated UTI is increasing off late. Extended Spectrum β-Lactamase (ESBL) production among UPEC strains pose a therapeutic challenge. As a result, the therapeutic options available for the treatment of UTI are cut down which in turn is linked to treatment failure and increase in the economic burden of the community. ^7^

Objectives: The study was undertaken to monitor the distribution of virulence factors among UPEC strains, to note the antibiogram, outcome, type of UTI and to look for the association of genetic virulence traits with antibiotic resistance.

## Methods

Study design: Prospective cross sectional time bound study.

Study setting: The study was conducted in the Department of Microbiology, in a tertiary care center at Mangalore, India, for a duration of six months (Study period: December 2020 to May 2021).

Participants: The inclusion and exclusion criteria are as mentioned below:

**Inclusion criteria:** All clinically significant isolates of *E.coli* from urine. An isolate was considered significant if urine cultures had colony count ≥10 ^5^ CFU/ml or ≥10 ^3^ CFU/ml in symptomatic patients. ^1^

**Exclusion criteria:** Urine samples with no growth, less significant counts of *E.coli*, growth of bacteria other than *E.coli,* isolates of *E.coli* from clinical samples other than urine.

The study was conducted after approval from the Institutional Ethics Committee, Kasturba Medical College, Mangaluru (Reg No. ECR/541/Inst/KA/2014/RR-17) Reference number IECKMCMLR-12/2020/408). The study was performed on isolates retrieved in the laboratory obviating the need for informed consent from the patients.

### Methodology

Specimen processing: Clean catch midstream urine samples received from suspected cases of UTI were processed within one hour of collection. The samples were inoculated onto MacConkey’s agar by semi quantitative method, Cystine-Lactose-electrolyte-deficient agar, UTI chrome agar. The culture plates were incubated for 37°C for 24 hrs. Urine samples with pure growth of *E.coli*, with a colony count of ≥10 ^5^ CFU/ml or ≥10 ^3^ CFU/ml in symptomatic patients, were considered significant. The study included 75 urinary isolates of *E. coli.* The isolates were identified based on colony morphology and standard biochemical tests. Antibiotic susceptibility testing was performed by the modified Kirby–Bauer disk diffusion/Vitek2 Compact (Biomerieux, France) system and interpretation was done as per the Clinical and Laboratory Standards Institute guidelines. ^8^

**Variables and data source:** The phenotypic methods for haemolysin production, serum resistance and genotypic characterization of virulence genes in UPEC are explained below.

**Phenotypic methods for detection of haemolysin production and serum resistance:**

The detection of α-haemolysin produced by *E. coli* was performed by plate haemolysis test. The presence of a zone of complete lysis of erythrocytes around the colony and clearing of the medium on 5% sheep blood agar, is suggestive of α-haemolysin production. ^9^^,^^10^

Serum resistance was studied by using fresh overnight culture of isolates as per the method described by Sharma *et al.* ^10^ The UPEC strains were considered serum sensitive if viable count dropped to 1% of the initial value and serum resistant if ≥90% of organisms survived after 180 minutes.

**Genotypic characterization of virulence genes of UPEC:** ^3^^,^^11^

DNA extraction was performed by boiling method. The spectrum of virulence genes in UPEC strains was detected using two sets of multiplex PCR as shown below. Table 1 shows the PCR mastermix preparation used. The primer sequence of the mentioned genes is shown in Table 2. PCR was performed in a final reaction volume of 50 μl. The program for amplification included a step of initial denaturation at 95°C for 3 min, followed by 25 cycles of 94°C for 30 s, 61°C for 30 s and 68°C for 3 min and a final extension step at 72°C for 3 min. The amplicons are visualized using the gel documentation system.

**Table 1.** PCR mastermix preparation for set 1 and set 2 of multiplex PCR.

Set 1: PCR assay for *papC* and *cnf1*		Set 2:PCR assay for *hlyA* and *iutA* genes	
**Template DNA**	4 μl	**Template DNA**	4 μl
**TaKaRa Taq’s 10X buffer (100 mM Tris-HCl, pH 8.9, MgCl2 +)**	5 μl	**TaKaRa Taq’s 10× buffer (100 mM Tris-HCl, pH 8.9, MgCl2 +)**	5 μl
**dNTP (0.2 mM each)**	4 μl	**dNTP (0.2 mM each)**	4 μl
***papC* primers (F and R)**	1.5 μl of 0.3 μM each	***hlyA* primers (F and R)**	3 μl of 0.6 μM each
***cnf1* primers (F and R)**	1.5 μl of 0.3 μM each	***iutA* primers (F and R)**	1.5 μl of 0.3 μM each
**Sterile PCR grade water**	33.8 μl	**Sterile PCR grade water**	30.8 μl
***Taq* DNA polymerase**	0.2 μl (1 U)	***Taq* DNA polymerase**	0.2 μl (1 U)
**Final reaction volume**	50 μl	**Final reaction volume**	50 μl

**Table 2.** Primer sequence of virulence genes in UPEC strains.

Virulence factor	Target gene(s)	Primer name	Primer Sequence (5′-3′)	Size of amplicon (bp)
Pilus assembly: central region of pap operon	*pap*C	*pap*C-f *pap*C-r	GTGGCAGTATGAGTAATGACCGTTA ATATCCTTTCTGCAGGGATGCAATA	200
The cytotoxic necrotizing factor I	*cnf*1	*cnf*1-F *cnf*1-R	AAGATGGAGTTTCCTATGCAGGAG CATTCAGAGTCCTGCCCTCATTAT T	498
α hemolysin	*hly*A	*hly*A-F *hly*A-R	AACAAGGATAAGCACTGTTCTGGCT ACCATATAAGCGGTCATTCCCGTCA	1177
Ferric aerobactin receptor	*iut*A	*iut*A-F *iut*A-R	ATCGGCTGGACATCATGGGAAC CGCATTTACCGTCGGGAACGG	300

The required data was retrieved from the clinical case records and the cases of complicated and uncomplicated UTI were identified. Uncomplicated UTIs are those that occur in healthy individuals without any of the predisposing factors for UTI. Complicated UTIs occur in individuals with underlying functional or structural abnormalities of the genitourinary tract. ^12^

**Sample size:** The sample size was calculated taking into account the data of the previously published article (2). Using the formula $n=\frac{Z^{2}\mathrm{pq}}{d^{2}}$ , a sample size of total 75 is calculated. Where *p*=prevalence=75%, *q*=1- *p*, *d*=Effect size=10%, *Z*=1.96 at 80-95% confidence interval.

The sample size for the study was 75.

### Statistical analysis

All the data was entered into an excel sheet and analyzed using IBM SPSS version 25. The continuous and categorical variables have been represented as mean ± standard deviation and frequency percentages respectively. The association between the variables were analyzed using the chi-square test.

## Results

A total of 75 urinary isolates of *E.coli* from patients with suspected urinary tract infections were included. Females had a higher preponderance of UTI (66.7%) than males (33.3%). Only 5 (7%) of the 75 patients were paediatric patients, remaining 70 (93%) were adults. The majority of the female patients were in the age group 20-39 years.

**Figure 1.**
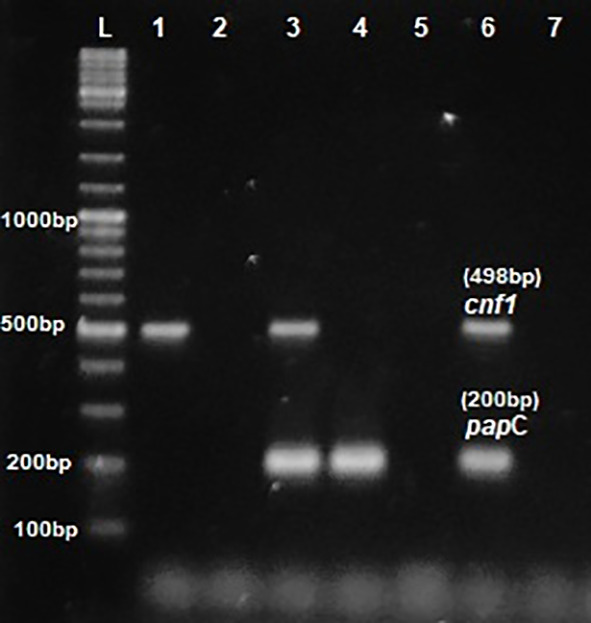
Multiplex PCR for *pap*C & *cnf*1. L- ladder 1000+ bp, lane 1-6: test isolates, lane 7: Negative control.

**Figure 2.**
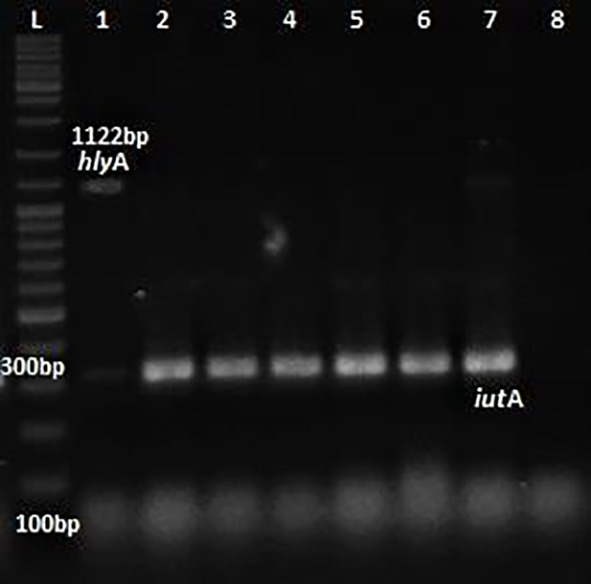
Multiplex PCR for *hly*A & *iut*A. L: ladder 1000+ bp, lane 1-7: test isolates, lane 8: Negative control.

**Figure 3.**
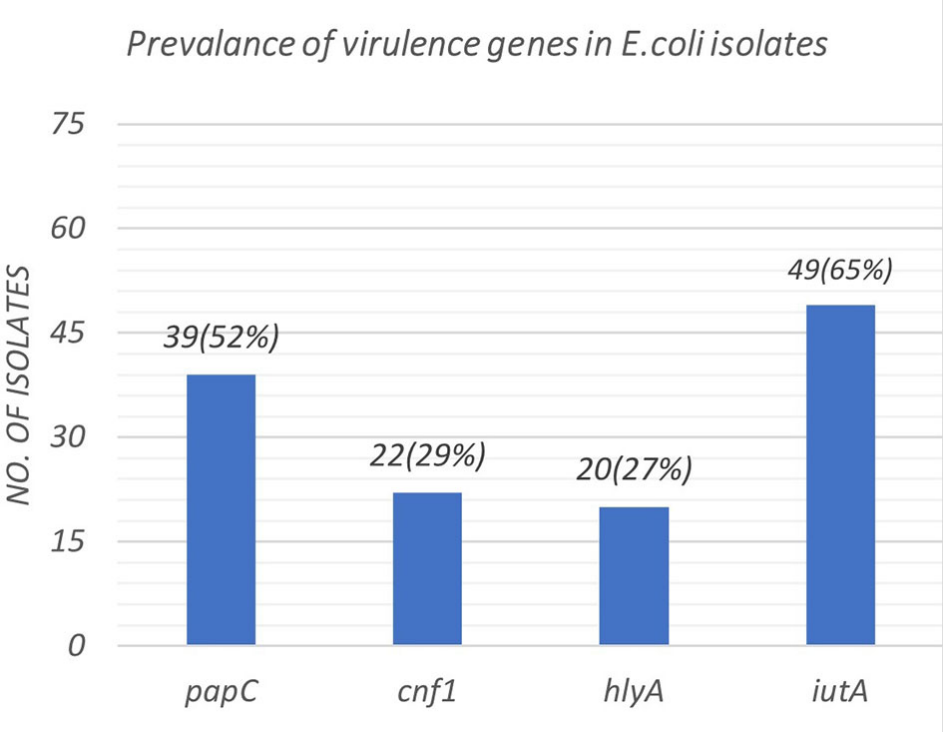
Prevalence of distribution of virulence genes among the 75 UPEC isolates.

Phenotypic detection of serum resistance and hemolysin production exhibited by *E.coli* was 100% and 32% respectively. Multiplex PCR was performed for the detection of virulence genes *pap*C, *Cnf*1, *hly*A & *iut*A as shown in Figures 1 and 2. The distribution of virulence genes among the 75 UPEC isolates is as shown in Figure 3. Out of 75 isolates, 65 were positive for at least one of the four targeted genes as shown in Table 3, while the remaining ten isolates were negative for all 4 genes.

**Table 3.** The spectrum of virulence genes detected in UPEC isolates.

No. of virulence genes detected	Virulence genes detected	No. of isolates
4	*pap*C *, cnf*1 *, hly*A *, iut*A	4
3	*pap*C *, cnf*1 *, hly*A	6
	*pap*C *, cnf*1 *, iut*A	7
	*pap*C *, hly*A *, iut*A	2
	*cnf*1 *, hly*A *, iut*A	1
2	*pap*C *, cnf*1	2
	*pap*C *, iut*A	12
	*cnf*1 *, iut*A	1
	*hly*A *, iut*A	4
1	*pap*C	5
	*cnf*1	1
	*hly*A	2
	*iut*A	18
Nil	Nil	10
Total		75

It was found that *hlyA* gene was found in a higher percentage in haemolytic isolates (41.7%) than non- haemolytic isolates (19.6%). p-value was statistically significant (p-value=0.044). Also, 50% of the *cnf*1 positive isolates harboured the *hly*A (haemolysin) gene (p=0.003; statistically significant).

**Figure 4.**
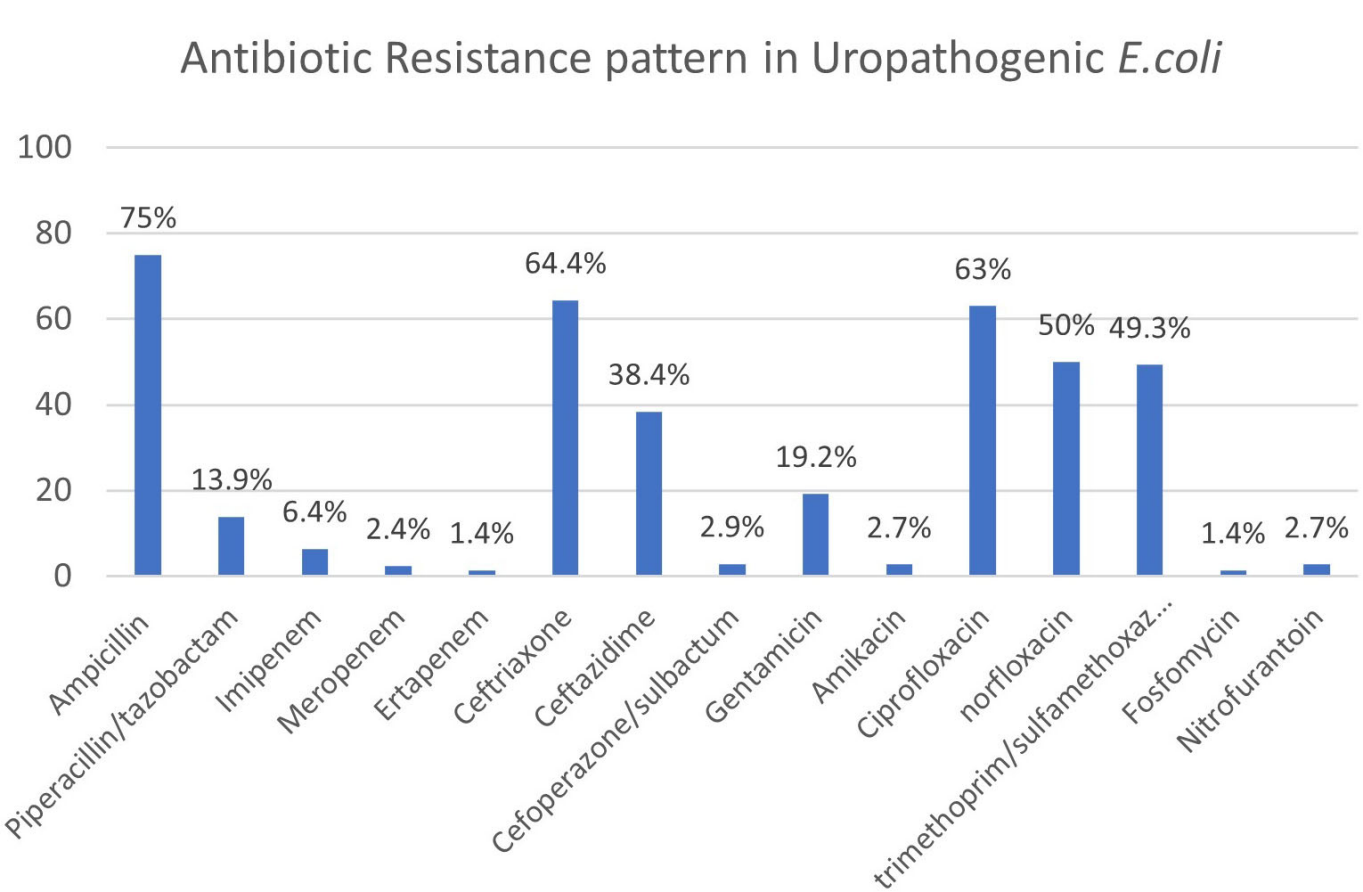
Resistance pattern in UPEC isolates.

The antibiotic resistance pattern of the UPEC isolates are as shown in Figure 4. Out of 75 isolates, 38 (50.6%) isolates were ESBL producers. Multidrug resistance (resistance to three or more antibiotic classes) was found in 40 (53.3%) isolates. Our study revealed that 45 out of the 75 urinary isolates, possessed more than one virulence factor as shown in Table 5.

The distribution of virulence factors in antibiotic-resistant isolates were studied as shown in Table 4. Statistical analysis (Chi square test ) for the association of the virulence factors detected and the resistance patterns of the isolates was done. Hemolysin production and resistance to imipenem, Norfloxacin was found to be significant (p≤0.05). The presence of *hlyA* gene and resistance to ceftazidime was found to be significant (p≤0.05). There was no statistically significant difference between the presence and absence of the other virulence genes with specific antibiotic resistance.

**Table 4.** Distribution of Virulence factors and genes in antibiotic-resistant isolate.

Virulence factors and antibiotic resistance		A	PiT	Ctr	Caz	Cfs	I	Etp	Mer	G	Ak	Nx	Cip	Fos	Nit
**Haemolysin**	**+ve (24)**	61.9% (21)	22.7% (5)	56.5% (13)	47.8% (11)	4.3% (1)	12.5% * (2)	4.8% (1)	-	21.7% (5)	4.2% (1)	19% (4) *	39.1% (9) *	4.2% (1)	4.3% (1)
	**-ve (51)**	80.9% (38)	10% (5)	66.7% (32)	34% (17)	2.1% (1)	-	-	2.1% (1)	18% (9)	-	61.7% (29) *	74% (37) *	-	2% (1)
**SR**	**+ve (75)**	75% (51)	13.9% (10)	63.4% (45)	38.4% (28)	2.9% (2)	4.3% (2)	1.4% (1)	2.1% (1)	19.2% (14)	1.4% (1)	48.5% (33)	63% (46)	1.4% (1)	2.7% (2)
***pap*C**	**+ve**	70.6% (24)	10.8% (4)	64.9% (24)	45.9% (17)	2.7% (1)	-	-	3.8% (1)	13.2% (5)	-	40% (14)	51.4% (19)	-	2.6% (1)
	**-ve**	79.4% (27)	19.1% (6)	61.8% (21)	30.6% (11)	3% (1)	8.7% (2)	3% (1)	-	25.7% (9)	2.9% (1)	57.6% (19)	75% (27)	2.9% (1)	2.9% (1)
***Cnf*1**	**+ve**	76.2% (16)	19% (4)	68.2% (15)	47.6% (10)	-	-	-	-	27.3% (6)		33.3% (7)	47.6% (10)	-	-
	**-ve**	74.5% (35)	11.8% (6)	61.2% (30)	34.6% (18)	4% (2)	6.1% (2)	2% (1)	3% (1)	15.7% (8)	2% (1)	55.3% (26)	69.2% (56)	2% (1)	3.9% (2)
***hly*A**	**+ve**	73.3% (11)	73.7% (14)	66.7% (12)	73.7% (14) *	5.3% (1)	-	-	-	26.3% (5)	-	33.3% (5)	63.2% (12)	5% (1)	-
	**-ve**	75.5% (40)	84.9% (45)	62.3% (33)	25.9% (14) *	2% (1)	6% (2)	2% (1)	2.8% (1)	16.7% (9)	1.9% (1)	52.8% (28)	63% (34)	-	3.8% (2)
***iut*A**	**+ve**	81.4% (35)	14.9% (7)	66.7% (30)	38.3% (18)	2.1% (1)	-	-	3.2% (1)	21.3% (10)	-	57.1% (24)	63.8% (30)	2.1% (1)	-
	**-ve**	64% (16)	12% (3)	57.7% (15)	38.5% (10)	4.3% (1)	15.4% (2)	4.2% (1)	-	15.4% (4)	3.8% (1)	34.6% (9)	61.5% (16)	-	7.7% (2)

A=Ampicillin, PiT=Piperacillin/tazobactam, Ctr=Ceftriaxone, Caz=Ceftazidime, Cfs=Cefoperazone/sulbactum, I=Imipenem, Etp=Ertapenem, Mer=Meropenem, G=Gentamicin, Ak=Amikacin, Nx=norfloxacin, Cip=Ciprofloxacin, Fos=Fosfomycin, Nit=Nitrofurantoin.

* p≤0.05 statistically significant.

Among the 40 MDR *E. coli* isolates, 11 (27.5%) isolates produced haemolysis and all were serum resistant. *iut*A, *pap*C, *cnf*1, *hly*A genes were detected in 72.5% (n=29), 47.5% (n=19), 35% (n=14) and 27.5% (n=11) of the MDR isolates respectively.

Out of the total 75 cases of UPEC studied, 60% (n=45) were complicated UTI and 40% (n=30) were uncomplicated UTI. Out of the complicated UTI cases, the common genes detected were *iut*A (83%) and *pap*C (50%) followed by *cnf*1 (33%) and *hly*A (10%) genes. Among the uncomplicated UTI isolates, *iut*A (77%), *pap*C (55%) and *cnf*1 (23%) were the common genes detected. None of the isolates from uncomplicated UTI had *hly*A. 77% of the complicated UTI were MDR and 33% of the uncomplicated UTI cases were MDR.

Out of the total 75 cases of UPEC studied, majority (97.4%) had a favourable clinical outcome at discharge. Catheter associated UTI was seen in 33.3% (n=25) of the patients. Mortality due to urosepsis was noticed in two cases (2.6%).

## Discussion

Urinary tract infection is one of the infectious diseases which is most prevalent amongst the people of all age groups from neonate to geriatric age group. Studies from India have reported varying prevalence rates of *E.coli* associated UTI- 50%-, 75%. ^13^ The bacterial pathogen accounting for the majority of community and hospital-acquired urinary infections is the UPEC. Depending on the virulence, these infections might range from mild uncomplicated UTI to complicated UTI. ^2^ Characterization of UPEC isolates with respect to their antibiotic resistance patterns and virulence factors, will aid in the effective management of UTI.

Our study revealed that 32% of the isolates produced haemolysin which is similar to the findings of Chhaya shah *et al* 34%, ^14^ but lower compared to the findings of Shetty *et al* (60%). ^15^ The exotoxin implicated in the enhanced virulence and lethality of clinical infections among UPEC strains is Alpha-hemolysin (Hly) production. ^16^ UTIs associated with Alpha-hemolysin may be associated with extensive kidney inflammation and injury due to it’s cytotoxic nature. The majority of *hly*A-positive strains were identified in patients with pyelonephritis (> 70%) compared to from patients with cystitis (31–48%), ascertaining the role of *hly*A as an important virulence factor in the pathogenesis of pyelonephritis. ^17^

Serum resistance is a property that provides a survival advantage to the bacteria. This makes UPEC resistant to killing by the alternative complement pathway in the normal human serum. Serum resistance in UPEC strains has been typically associated with pyelonephritis, cystitis and bacteraemia. ^7^ In the current study, all 75 isolates showed resistance to serum bactericidal action. This is similar to the studies by Sharma *et al* and Shetty SK *et al* which reported 86.7% and 83% of serum resistance. ^10^^,^^18^ However, study by Anuradha *et al* revealed 51% of serum resistance. ^7^ In UPEC, capsule and the O antigen are polysaccharides that contribute to virulence. The extracellular polysaccharides are antiphagocytic and inhibit complement-mediated lysis. ^19^ All the strains in this study on UPEC showed serum resistance indicating its significant association with UTI.

In the current study, the genes coding for adherence gene (papC), Cytotoxic necrotizing factor I ( *cnf*1), α haemolysin ( *hly*A) and ferric aerobactin receptor ( *iut*A) were detected by multiplex PCR.

The virulence gene *iut*A, is the gene for the siderophore ferric aerobactin receptor which helps the bacterial intake of iron and this enables the survival of bacteria in an atmosphere with limited concentrations of iron (urinary tract) This gene was the commonest gene detected in the UPEC isolates of our study(65%), thus proving its association with the virulence of UPEC. This finding supports the data published by Munkhdelger *et al* (62.2%), ^20^ and Karam *et al* (66.4%). ^21^

The rate of detection of adherence gene ( *pap*C) was 52%. This is in par with a study by Chakraborty A *et al* in 2017 in which nearly half of their isolates (49%) carried this gene. ^3^ Firoozeh *et al* reported a lower rate of detection (34.6%) of *pap*C gene in UPEC isolates. ^22^

27% of our isolates were positive for *hly*A gene which was similar to the study by Gohar *et al* (26%). ^23^ A lower rate (13.6%) of *hly*A gene were seen by Daniela A *et al* study. ^24^

Toxigenic strains of *E.coli* like necrotoxigenic *E.coli-1*(NTEC-1) produce Cytotoxic necrotizing factor 1 ( *cnf*1). ^25^ Our study showed that 29%(n=33) were NTEC -1 strains harbouring the gene *cnf1.* The distribution of *cnf*1 in our study is similar to the studies by Chakraborty A *et al* (29.5%) and Gohar *et al* (30%). ^3^^,^^23^ NTEC -1 strains are emerging pathogens in India. ^25^ Our study revealed that 50% of the *cnf*1 positive isolates harboured *hly*A (haemolysin) gene, which is statistically significant. The combined production of several powerful toxins (haemolysin, CNF) and co-expression of various virulence genes by NTEC strains makes them potentially aggressive pathogens. ^25^ Hence identification of these strains at an early stage, would prevent the complications associated with NTEC -1 strains.

The virulence genes studied are involved in the pathogenesis of UTI. However the absence of genes in 10 isolates (13.3%) could possibly be due to mutation of the gene Thus, a negative PCR result doesnot rule out the absence of virulence genes.

Varying spectrum and rates of antibiotic resistance have been reported among the UPEC isolates and most studies have reported that amikacin, nitrofurantoin and imipenem are highly efficacious against such strains. ^21^ In our study, a high percentage of resistance was noted to beta lactam group of antibiotics (ampicillin-75%, ceftriaxone-64.4%), fluoroquinolones (ciprofloxacin-63%, norfloxacin-50%), trimethoprim/sulfamethoxazole (49.3%). Lower rates of resistance was detected with respect to piperacillin/tazobactam(13.9%), carbapenems (ertapenem-1.4%, imipenem-6.4%), amikacin(2.7%) and nitrofurantoin (2.7%), Cefoperazone/sulbactam (2.9%) and fosfomycin (1.4%). Based on these findings, nitrofurantoin, piperacillin tazobactam and cefaperazone sulbactam maybe suitable candidates for empirical therapy of UTIs. However, the therapeutic utility of these drugs in complicated UTI in comparison with uncomplicated UTI was not studied. Drugs like aminoglycosides, carbapenems and fosfomycin maybe used as reserve drugs in the management of MDR-UTI due to *E. coli.*

Nitrofurantoin being an oral drug maybe a good drug of choice in such patients with UTI and outpatients. These results are in par with a study which revealed that nitrofurantoin and carbapenems are suitable as empirical antibiotics in the treatment of UTIs in outpatients and fosfomycin can be used against highly resistant UPEC isolates. ^26^ However, their inappropriate usage may gradually give rise to their increase in antibiotic resistance. This accentuates the need for local hospital data compilation and antibiotic resistance analysis to devise a suitable antibiotic policy for the hospitals.

We found that 51% were ESBL producers which is similar to studies by Chhaya *et al* (46%) & Shoba *et al* (19%-59.6%). ^14^^,^^27^ In our study, 45 isolates expressed multiple virulence factors ( Table 5), among which a majority (57.7%) were non ESBL producers. These results support the fact that the expression of virulence genes maybe inversely related to the presence of antibiotic resistance and ESBL production.

**Table 5.** Presence of multiple virulence factors in ESBL producing isolates of *E.coli.*

Multiple virulence factors (no. of isolates)	ESBL +	ESBL -
+ (45)	19	26
_ (30)	19	11
Total 75	38	37

The distribution of virulence factors in antibiotic-resistant isolates showed that hemolysin production is significantly associated with resistance to imipenem, norfloxacin and ciprofloxacin (p≤0.05) at par with findings of Chhaya *et al.* ^14^ The presence of *hlyA* gene is significantly associated with resistance to Ceftazidime (p≤0.05). The association of the presence of *hly*A with resistance to third generation cephalosporins poses a therapeutic challenge. ^28^ No other statistically significant difference was proved between the presence or absence of the other virulence factors, with any specific antibiotic resistance.

Multidrug resistance was observed in 40 isolates (53.3%). This finding is comparable to the study by Hasan *et al* ^29^ in India, in which the prevalence of MDR E. coli was 52.9%. However, this finding is in contrast to study by Munkhdelger *et al* ^21^ which revealed a higher rate of MDR in UPEC (93.9%).

In our study, 27.5% (n=11) MDR isolates produced haemolysis and all the 40 MDR isolates showed serum resistance. Co-existence of MDR with hemolysis or serum resistance may contribute to the increased pathogenicity and nonresponse to therapy in cases of UPEC associated UTI. In our study, *iut*A, *pap*C, *cnf*1, *hly*A genes were detected in 72.5% (n=29), 47.5% (n=19), 35% (n=14) and 27.5% (n=11) of the MDR isolates respectively. *iut*A was the commonest gene detected in MDR isolates. This finding supports the fact that certain virulence genes like *iut*A is positively associated with MDR. ^30^

Inappropriate use of broad-spectrum antibiotics, prolonged hospitalisation, poor hygiene are few major factors that contribute to increasing MDR infections. In our research, the percentage of MDR in complicated UTI was 77%; which was higher compared to uncomplicated UTI (33%). Our study supports the findings of a study by Johnson J R *et al,* which revealed that antibiotic resistance was higher in people with complicated UTIs than with uncomplicated UTIs. ^31^

In our study, third generation cephalosporins (n=15, 37.5%), meropenem (n=12, 30%), piperacillin tazobactam (n=8, 20%) and nitrofurantoin (n=5, 12.5%) were the antibiotics used in the treatment of these MDR cases.

Out of the total 75 cases of UPEC studied, majority i.e 60% (n=45) were cases of complicated UTI. Distribution of *cnf*1 gene was more in complicated UTIs compared to uncomplicated UTIs. *hly*A gene detected in complicated UTIs was absent in isolates from uncomplicated UTIs. This difference in results, regarding the distribution of *hly*A and *cnf*1 genes, maybe attributed to the fact hemolysin production and *cnf*1 are attributed to the dysfunction of local immune response and host tissue damage. Thus, probably there is increased expression of *hly*A and *cnf*1 in complicated UTIs. There is no significant difference in the distribution of genes associated with adhesion ( *pap*C) and iron uptake ( *iut*A). This finding is in contrast to the study by Takahashi *et al*, which revealed that the prevalence of *pap*C was more in cases of uncomplicated UTI. ^32^

The three isolates from patients with history of emphysematous pyelonephritis and urosepsis, were found to be positive for virulence genes *pap*C (adherence) and *cnf*1 (cytotoxicity). *cnf*1 gene is associated with extensive tissue damage ^4^ and this could have contributed to the emphysematous pyelonephritis in these three cases.

Mortality due urosepsis was noticed in two cases (2.6%). Isolates of both these cases had all the 4 genes, produced hemolysin, were serum resistant and MDR. This substantiates the contribution of hemolysin production, serum resistance and expression of the virulence genes in the increased pathogenicity of UPEC.

Limitations of the study were that the study could have been performed on a larger sample size for better characterization of UPEC. The other limitation would be that the authors could not compare the association of the comorbidities such as immunocompromised condition and exposure to frequent antibiotics to look for any differences in the antibiotic resistance patterns, since the virulence characteristics are different among those populations. One more limitation was that data on nosocomial/community acquired UTI was not studied.

## Conclusion

The association of hemolysin production with resistance to imipenem and norfloxacin in UPEC strains was found to be significant and the presence of *hlyA* gene is positively associated with ceftazidime resistance, thus posing a therapeutic challenge. Nitrofurantoin, piperacillin tazobactam, cefoperazone/sulbactam, carbapenems, fosfomycin and amikacin were the antibiotics against UPEC which showed lower rates of resistance. Owing to the lower rate of resistance and ease of administration, nitrofurantoin maybe an effective oral drug in outpatients with UTI. While Piperacillin tazobactam and cefaperazone sulbactam maybe suitable for empirical therapy of UTIs in inpatients. Drugs like aminoglycosides, carbapenems and fosfomycin may be used as reserve drugs in the treatment of MDR-UTI. However, inappropriate usage can gradually increase antibiotic resistance. Hence, proper selection of antibiotics in hospitals taking into account the local antibiogram is needed to reduce the emergence of antibiotic resistance. The data on distribution of virulence factors and antibiotic resistance helps in planning the management strategies in UTI in our setup, thus improving patient care.

## Data availability

Dryad. Characterization of virulence factors and antibiotic resistance pattern of Uropathogenic *Escherichia coli* strains in a tertiary care center. The full reference for data repository provided by dryad for access is as follows: DOI: https://doi.org/10.5061/dryad.q83bk3jmd. ^33^

## Author contributions

1. Mr Naveen Kumar M: Conceptualization, Data Curation, Formal Analysis, Investigation, Methodology, SoftwareSupervision, Validation, Visualization, Writing & Editing
2. Dr Sevitha Bhat: Data Curation, Formal Analysis, Investigation, Methodology, Project Administration, Supervision, Validation, Visualization, Writing – Review & Editing
3. Dr Archana Bhat K - Conceptualization, Data Curation, Formal Analysis, Investigation, Methodology, ProjectAdministration, Resources, SoftwareSupervision, Validation, Visualization, Writing – Review & Editing
4. Dr Shalini Shenoy M: Conceptualization, Project Administration, Resources, Supervision, Writing – Review & Editing
5. Dr Vishwas Saralaya: Conceptualization, Project Administration, Resources, Supervision, Writing.
