# Supplementary material for: Characterization of virulence factors and antibiotic resistance pattern of uropathogenic Escherichia coli strains in a tertiary care center
Source: F1000Res. 2022 Nov 28;11:1163. Originally published 2022 Oct 11. [Version 2] doi: 10.12688/f1000research.125596.2 (PMC9723409; doi:10.12688/f1000research.125596.2)
Supplement: Peer review history

# Peer review: Reviewer response for version 2

**DOI:** 10.5256/f1000research.141529.r156819

**Published:** 2022-12-05

**Authors:** Vignesh Ramachandran

**Affiliations:** 1 Faculty of Medicine, Universiti Kuala Lumpur-Royal College of Medicine Perak, Ipoh, Malaysia

The comments raised have been appropriately addressed in the revised manuscript.

Is the work clearly and accurately presented and does it cite the current literature?

Yes

If applicable, is the statistical analysis and its interpretation appropriate?

Yes

Are all the source data underlying the results available to ensure full reproducibility?

Yes

Is the study design appropriate and is the work technically sound?

Yes

Are the conclusions drawn adequately supported by the results?

Yes

Are sufficient details of methods and analysis provided to allow replication by others?

Yes

Reviewer Expertise:

Medical Microbiology

I confirm that I have read this submission and believe that I have an appropriate level of expertise to confirm that it is of an acceptable scientific standard.

# Peer review: Reviewer response for version 1

**DOI:** 10.5256/f1000research.137922.r154567

**Published:** 2022-11-17

**Authors:** Anusha Rohit

**Affiliations:** 1 Division of Infectious Diseases, Nitte University Centre for Science Education and Research (NUCSER), Nitte (Deemed to Be University), Mangalore, Karnataka, India; 2 The Madras Medical Mission, Chennai, India

The manuscript describes the analysis of 75 isolates of UPEC for virulence factors and antimicrobial resistance. The virulence factors and their association with resistance was also discussed. The design of the study was prospective cross-sectional.

- As 60% of the isolates were from complicated UTIs, can the authors explain why they suggest nitrofurantoin for therapy in the conclusion of the paper?
- Was any statistical analysis done for the association of the virulence factors detected and the resistance patterns of the isolates?
- Why did the authors choose these genes for the detection of the virulence characterisation as many of these characteristics have multiple genes coding for them? The association between *pap*C *, iut*A seemed to be the most common among the isolates.
- How many of these isolates were from patients who developed CAUTI due to the presence of a urinary catheter?

Is the work clearly and accurately presented and does it cite the current literature?

Yes

If applicable, is the statistical analysis and its interpretation appropriate?

Yes

Are all the source data underlying the results available to ensure full reproducibility?

Yes

Is the study design appropriate and is the work technically sound?

Yes

Are the conclusions drawn adequately supported by the results?

Yes

Are sufficient details of methods and analysis provided to allow replication by others?

Yes

Reviewer Expertise:

Antimicrobial resistance, infection control

I confirm that I have read this submission and believe that I have an appropriate level of expertise to confirm that it is of an acceptable scientific standard.

# Response

**Published:** 2022-11-24

**Authors:** Archana Bhat K

**Affiliations:** KMC Mangalore, India

As per reviewer suggestions, the following changes will be done accordingly and updated in the revised text of the manuscript and uploaded.

 1. As 60% of the isolates were from complicated UTIs, can the authors explain why they suggest nitrofurantoin for therapy in the conclusion of the paper?

- The authors have updated the discussion and conclusion in the paper . Owing to the lower rate of resistance and ease of administration, nitrofurantoin maybe an effective oral drug in outpatients with UTI (added in conclusion). However, the therapeutic utility of nitrofurantoin in complicated UTI compared to uncomplicated UTI was not studied.(added in discussion)

2. Was any statistical analysis done for the association of the virulence factors detected and the resistance patterns of the isolates?

- Statistical analysis (Chi square test ) for the association of the virulence factors detected and the resistance patterns of the isolates was done .The authors have added a sentence on the same. The results of significance is already mentioned in the content of discussion with reference to table 4.

3. Why did the authors choose these genes for the detection of the virulence characterisation as many of these characteristics have multiple genes coding for them? The association between *pap*C *, iut*A seemed to be the most common among the isolates.

- The authors have updated the content in Background of the study-“Based on previously published literature, we aimed to look into the common genes *pap*C and *iutA* and apart from them, other genes *hly *A and *cnf1* were screened for epidemiological and virulence characterisation.”

4. How many of these isolates were from patients who developed CAUTI due to the presence of a urinary catheter?

- The authors have updated the results and added the sentence “Catheter associated UTI was seen in 33.3%(n=25) of the patients.”

# Peer review: Reviewer response for version 1

**DOI:** 10.5256/f1000research.137922.r153296

**Published:** 2022-10-31

**Authors:** Vignesh Ramachandran

**Affiliations:** 1 Faculty of Medicine, Universiti Kuala Lumpur-Royal College of Medicine Perak, Ipoh, Malaysia

The authors have planned, executed, and presented an important piece of work and the information from the findings adds so much value to the medical literature. The study findings are very significant and hope a much larger study will be planned based on these results.

 However, there are a few minor comments to be addressed by the authors.

1. The first line of the abstract and introduction part begins with, " *Urinary tract infections (UTI)* *are the most prevalent bacterial infections*". This statement is too strong and needs scientific backing. It would be appropriate to say "one of the common bacterial infections".
2. The references cited in numbers one and two are not from properly indexed journals and it would be better to cite references from reputed journals.
3. Reference number 31 is not cited properly - need to include the journal's name.
4. In the methodology section, need to give a reference for the CLSI method.
5. The second line of the results section starts with a numeral (93%), it has to start with the text.
6. There is no clear mention of the demographic characteristics of the study population. From the results, it is evident that the population is mixed including pediatrics as well as adults. It would add value to know about the study population. What if there were immunocompromised patients included and exposed to frequent antibiotics, the resistance patterns would be different in them. Moreover, the virulence characteristics are also different among those populations. How did the authors rule out other comorbidities and immune effects in the study population?
7. How many of the cases were community-acquired and how many were nosocomial? These factors have major implications in affecting the study results.

Is the work clearly and accurately presented and does it cite the current literature?

Yes

If applicable, is the statistical analysis and its interpretation appropriate?

Yes

Are all the source data underlying the results available to ensure full reproducibility?

Yes

Is the study design appropriate and is the work technically sound?

Yes

Are the conclusions drawn adequately supported by the results?

Yes

Are sufficient details of methods and analysis provided to allow replication by others?

Yes

Reviewer Expertise:

Medical Microbiology

I confirm that I have read this submission and believe that I have an appropriate level of expertise to confirm that it is of an acceptable scientific standard, however I have significant reservations, as outlined above.

# Response

**Published:** 2022-11-24

**Authors:** Archana Bhat K

**Affiliations:** KMC Mangalore, India

As per reviewer's suggestions, the changes mentioned below will be done accordingly in the updated text file of the manuscript and submitted to the journal.

**1. The first line of the abstract and introduction part begins with, "Urinary tract infections (UTI) are the most prevalent bacterial infections". This statement is too strong and needs scientific backing. It would be appropriate to say "one of the common bacterial infections".**

 Changes have been done in the first line of the abstract and introduction part as per reviewer suggestions.

**2. The references cited in numbers one and two are not from properly indexed journals and it would be better to cite references from reputed journals.**

 The references 1 and 2 have been updated.

 3. **Reference number 31 is not cited properly - need to include the journal's name.**

 The journal's name has been added and updated in references and citation.

 4. **In the methodology section, need to give a reference for the CLSI method.**

 In the methodology section, reference for the CLSI method has been cited. (Reference 8 ) References have been updated in serial order thereon.

 5. T **he second line of the results section starts with a numeral (93%), it has to start with the text.**

 Modified the second line of the results section.

 6. **There is no clear mention of the demographic characteristics of the study population. From the results, it is evident that the population is mixed including pediatrics as well as adults. It would add value to know about the study population. What if there were immunocompromised patients included and exposed to frequent antibiotics, the resistance patterns would be different in them. Moreover, the virulence characteristics are also different among those populations. How did the authors rule out other comorbidities and immune effects in the study population?**

 Included this as a limitation of the study in the last paragraph of discussion.

**7. How many of the cases were community-acquired and how many were nosocomial? These factors have major implications in affecting the study results**

 Included this as a limitation of the study in the last paragraph of discussion.
